# Research on Bearing Fault Diagnosis Method for Varying Operating Conditions Based on Spatiotemporal Feature Fusion

**DOI:** 10.3390/s25123789

**Published:** 2025-06-17

**Authors:** Jin Wang, Yan Wang, Junhui Yu, Qingping Li, Hailin Wang, Xinzhi Zhou

**Affiliations:** 1 School of Electronic Information, Sichuan University, Chengdu 610065, China; jin_w6688@163.com (J.W.); wyxst@163.com (Y.W.); yujh2000@126.com (J.Y.); lina_lqp@163.com (Q.L.); 2 National Key Laboratory of Science and Technology on Reactor System Design Technology, Nuclear Power Institute of China, Chengdu 610213, China; hailin_lie@163.com

**Keywords:** spatiotemporal features, attention mechanism, unsupervised domain adaptation, transfer learning

## Abstract

In real-world scenarios, the rotational speed of bearings is variable. Due to changes in operating conditions, the feature distribution of bearing vibration data becomes inconsistent, which leads to the inability to directly apply the training model built under one operating condition (source domain) to another condition (target domain). Furthermore, the lack of sufficient labeled data in the target domain further complicates fault diagnosis under varying operating conditions. To address this issue, this paper proposes a spatiotemporal feature fusion domain-adaptive network (STFDAN) framework for bearing fault diagnosis under varying operating conditions. The framework constructs a feature extraction and domain adaptation network based on a parallel architecture, designed to capture the complex dynamic characteristics of vibration signals. First, the Fast Fourier Transform (FFT) and Variational Mode Decomposition (VMD) are used to extract the spectral and modal features of the signals, generating a joint representation with multi-level information. Then, a parallel processing mechanism of the Convolutional Neural Network (SECNN) based on the Squeeze-and-Excitation module and the Bidirectional Long Short-Term Memory network (BiLSTM) is employed to dynamically adjust weights, capturing high-dimensional spatiotemporal features. The cross-attention mechanism enables the interaction and fusion of spatial and temporal features, significantly enhancing the complementarity and coupling of the feature representations. Finally, a Multi-Kernel Maximum Mean Discrepancy (MKMMD) is introduced to align the feature distributions between the source and target domains, enabling efficient fault diagnosis under varying bearing conditions. The proposed STFDAN framework is evaluated using bearing datasets from Case Western Reserve University (CWRU), Jiangnan University (JNU), and Southeast University (SEU). Experimental results demonstrate that STFDAN achieves high diagnostic accuracy across different load conditions and effectively solves the bearing fault diagnosis problem under varying operating conditions.

## 1. Introduction

With the continuous advancement of modern intelligent technologies and the rapid development of big data in industrial equipment, ensuring the safety of machinery has gradually become one of the key factors affecting economic development and social progress. The daily maintenance and fault prediction of complex mechanical systems have become increasingly important. Bearing fault diagnosis plays a crucial role in the timely detection of equipment failures and ensuring normal operation. Improving the accuracy of bearing fault diagnosis is of significant importance in preventing disasters and ensuring personal safety [1].

Traditional machine learning methods still require manual selection of valuable features for fault classification, which may lead to the loss of potential information. With the development of artificial intelligence technologies, data-driven deep learning methods have emerged. Deep learning techniques can automatically extract features for subsequent fault identification without human intervention, often outperforming traditional methods and improving fault detection accuracy [2]. Eren et al. [3] proposed a bearing fault diagnosis method based on a compact adaptive 1D convolutional neural network (1D-CNN). This method trains the network to learn optimal features and demonstrates its effectiveness on two vibration datasets. Pham et al. [4] introduced a fault diagnosis method combining an improved Generative Adversarial Network (GAN) with 2D representations of acoustic emission signals. By converting 1D signals into 2D images, the method preserves the time-frequency information of the signals, facilitating feature extraction. Li et al. [5] proposed a framework based on deep neural networks (DNN), integrating time-series models such as LSTM or GRU to handle dynamic characteristics. Gong et al. [6] developed a multi-channel deep convolutional neural network (MC-DCNN) model, aiming to integrate vibration and current signals from motors for effective composite fault diagnosis. Wen et al. [7] transformed 1D time-domain fault signals into RGB image format to meet the input requirements of ResNet-50, utilizing the pre-trained ResNet-50 model on ImageNet to extract deep features from the input signals.

Deep learning-based fault diagnosis requires two fundamental conditions to be met: (1) the source domain (training set) and target domain (test set) must follow the same data distribution. However, in real industrial environments, the operational states of machines are not static, and there is often a distribution shift between the source and target domains. While deep learning methods can automatically learn deep features from raw data without human intervention, their ability to generalize across different but related tasks is not guaranteed. (2) There must be sufficient labeled fault data in the target domain. In many industrial applications, the proportion of labeled samples in large-scale datasets is relatively small. Due to cost and expertise limitations, it is nearly impossible to label all data. Without sufficient labeled data for training, these methods become ineffective, which significantly limits the application of deep learning-based intelligent fault diagnosis methods in relevant industries. To address the shortcomings of deep learning in handling real-world data, deep transfer learning methods have emerged, combining deep learning and transfer learning. This approach significantly enhances the model’s generalization ability and accuracy, especially when there is a lack of labeled data in the target domain.

Deep Transfer Learning (DTL) can apply the knowledge learned from labeled source domain data to the unlabeled target domain through unsupervised domain adaptation techniques, thus enabling the diagnosis of unknown faults in the target domain using feature relationships from the source domain. According to [8], transfer learning methods include network-based DTL, instance-based DTL, mapping-based DTL, and adversarial DTL. Tian et al. [9] proposed a self-adaptive label filtering learning method for unsupervised domain adaptation. By designing a graph-based random walk strategy to predict pseudo-labels and refining them through a self-adaptive label filtering mechanism, the model’s performance in the target domain is improved. Wang et al. [10] proposed a hierarchical deep domain adaptation method for fault diagnosis, which calculates marginal and conditional correlation alignment metrics for each layer of a stacked denoising autoencoder to achieve fault diagnosis under different operating conditions. Zheng et al. [11] proposed a progressive multi-source domain adaptation method aimed at enhancing the generalization ability of diagnostic models. Particularly in cases where multi-source domain data exhibit distribution discrepancies, the model can more effectively learn features relevant to the target domain by gradually adapting to the data distributions of multiple source domains. Yang et al. [12] proposed a joint distribution alignment strategy that combines the attention mechanism with Joint Adaptation Network (JAN), which can effectively tap the migration features between source and target domains. Li et al. [13] used Joint Maximum Mean Difference (JMMD) to align the joint distribution of source and target domains in Joint Adaptation Network (JAN), which effectively reduces the domain bias and enhances the statistical matching by minimizing the domain classification loss through the domain classifiers in the adversarial network. Guo et al. [14] proposed a Deep Discriminative Adversarial Domain Adaptive Neural Network (DDADAN) that preprocesses the vibration signals by Fast Fourier Transform and extracts the vibration signals using an improved wide first layer convolutional neural network to extract deep features, and the distributional alignment of source and target domains is realized by combining the correlation alignment (CORAL) algorithm and the adversarial training mechanism. Li et al. [15] proposed a MixStyle network based on the SE attention mechanism combined with Bulk Spectrum Penalty (BSP) to enable the network to automatically focus on key features, increase the source domain data diversity and other feature vectors, and improve the feature discriminative power and domain adversarial performance. Yang et al. [16] proposed a domain adaptation model based on multiscale residual network to extract the multiscale features of vibration signals through the multiscale module, combined with the APReLU activation function to adaptively adjust the nonlinear transformations, and finally realized the cross-domain fault diagnosis of aero-engine bearings through the joint distribution of the aligned source and target domains by JMMD.

Understanding the structural information in data helps reduce the distribution discrepancy, thereby improving the transferability between the source and target domains. To understand the data structure, data processing methods such as adaptive modal decomposition [17], empirical mode decomposition [18], variational mode decomposition [19], short-time Fourier transform [20], and wavelet transform [21] have been studied in the field of fault diagnosis and transfer learning. Kuo et al. [22] used Wavelet Transform to convert vibration signals into time-frequency images, and employed a deep learning model to perform fault diagnosis on machine tools under varying operating conditions. This method significantly improved diagnostic performance by transferring knowledge from the source domain to the target domain. Tong et al. [23] proposed a domain-adaptation-based fault diagnosis method by preprocessing vibration signals with Fast Fourier Transform (FFT) to obtain datasets under different motor speeds and load conditions. They reduced the distribution discrepancy using Maximum Mean Discrepancy (MMD), thus enabling fault diagnosis under varying conditions.

Among these, mapping-based and adversarial methods require feature extractors to first extract features, which are then aligned between the source and target domains using transfer learning techniques. The concept of spatiotemporal feature extraction from input data has already been proposed. Peng et al. [24] introduced a fault diagnosis method based on spatiotemporal clustering and a deep attention subdomain adaptive residual network. By employing a clustering algorithm to extract spatiotemporal features from the vibration signals of pitch bearings, this method captures the spatial distribution and temporal evolution characteristics of fault signals, thereby improving the accuracy and robustness of pitch bearing fault diagnosis. Guo et al. [25] proposed a method using 1D Convolutional Neural Networks (1D-CNN) to automatically learn features and identify the health status of equipment. By maximizing domain recognition errors and minimizing the probability distribution distance, this approach encourages the 1D-CNN to learn domain-invariant features, enabling knowledge transfer from the source domain to the target domain. Khorram et al. [26] proposed an end-to-end deep learning method combining CNN and LSTM for bearing fault diagnosis. This approach directly uses raw time-domain vibration signals collected by accelerometers as input for fault diagnosis. However, for fault diagnosis under different conditions, in the serial model, the features extracted from the CNN need to be gradually passed to the BiLSTM for processing, which ultimately results in the temporal relationship of the extracted temporal features describing the spatial features, leading to the loss of potential temporal information in the original vibration data. In contrast, the parallel model can simultaneously utilize the advantages of both models for feature extraction. By choosing an appropriate feature fusion method, the extracted features can be efficiently integrated to provide greater flexibility and usability. This enhances the ability of the model to learn from complex data and avoid such bottlenecks.

In summary, to address the issue of bearing fault diagnosis under varying operating conditions, this paper proposes a parallel processing network. Compared to previous serial networks, the proposed parallel network preserves the independence of each network’s role. First, a data augmentation method combining Fast Fourier Transform (FFT) and Variational Mode Decomposition (VMD) is used to extract time-frequency features from vibration signals. Then, parallel modeling is performed using a Squeeze-and-Excitation-based Convolutional Neural Network (SECNN) and a Bidirectional Long Short-Term Memory network (BiLSTM) to capture both spatial and temporal dependencies. A cross-attention mechanism is employed to efficiently fuse the spatiotemporal features. Finally, Multi-Kernel Maximum Mean Discrepancy (MKMMD) is applied to align the feature distributions between the source and target domains, solving the fault diagnosis problem under varying operating conditions. The main contributions of this paper are summarized as follows:

(1) A data processing method combining Fast Fourier Transform (FFT) and Variational Mode Decomposition (VMD) is proposed. Compared to traditional methods using only FFT or VMD, this approach enhances the diversity and richness of data representation by extracting frequency-domain features with FFT and local modal features with VMD, effectively improving the information representation.
(2) This paper proposes a parallel processing spatiotemporal feature fusion domain-adaptive network (STFDAN), which combines the parallel network structure with MKMMD to build a high-accuracy bearing fault diagnosis model under varying operating conditions. Compared to traditional serial frameworks, the parallel architecture preserves the uniqueness of both components, enhancing the model’s adaptability to multimodal data or complex feature distributions. It avoids information loss caused by the mixing of different modal features in serial structures, improving feature diversity and representation efficiency.
(3) The effectiveness and superiority of the proposed model in fault diagnosis under varying operating conditions are demonstrated using publicly available bearing datasets from Case Western Reserve University, Southeast University, and Jiangnan University. A comparison with traditional transfer learning methods is also provided.

The rest of the paper is structured as follows: Section 2 briefly introduces the detailed structure and components of the proposed algorithm. Section 3 presents sufficient case studies and process analysis for fault diagnosis under varying operating conditions, validating the performance of the Spatiotemporal Feature Fusion Domain-Adaptive Network (STFDAN) in fault diagnosis under varying conditions. Finally, Section 4 provides a comprehensive conclusion.

## 2. Proposed Method Framework

### 2.1. STFDAN Model Framework

**Figure 1:**
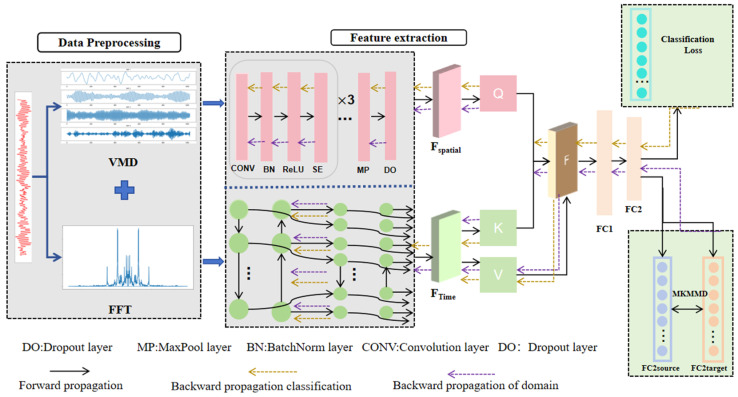
STFDAN Model Architecture.

In this section, we propose a Spatiotemporal Feature Fusion Domain-Adaptive Network (STFDAN) to address the problem of fault diagnosis for bearings under varying operating conditions. The architecture of the STFDAN model is shown in Figure 1.

First, labeled data from the source domain and unlabeled data from the target domain undergo FFT and VMD decomposition to extract multi-scale time-frequency information from the signals. These features are then normalized to eliminate scale differences and enhance representational consistency. A dual-path modeling approach is adopted by combining SECNN and BiLSTM. The SECNN leverages a channel attention mechanism to dynamically regulate the importance of frequency and temporal channels, enhancing the selection of critical features and generating efficient local spatial representations. Meanwhile, BiLSTM employs bidirectional temporal modeling to deeply explore dynamic correlations in the sequence, capturing the long-term dependencies of temporal evolution, thus forming global temporal representations. To address the challenge of weight allocation for spatiotemporal feature fusion, a cross-attention mechanism is introduced. This mechanism constructs consistent query, key, and value vector spaces, facilitating information enhancement and mutual compensation across feature dimensions, ensuring the comprehensiveness and efficiency of semantic interaction between features. For domain adaptation, Multi-Kernel Maximum Mean Discrepancy (MKMMD) is used to align the feature distributions of the source and target domains with high precision. The classification loss and alignment loss are jointly optimized in the objective function. Through backpropagation, the network parameters are dynamically adjusted, enabling the feature extraction module to generate domain-invariant representations and maximize the generalization capability of knowledge transfer from the source domain.

### 2.2. Data Augmentation

The Fast Fourier Transform (FFT) is an efficient algorithm for performing Fourier transformations on time-domain signals, breaking them down into different frequency sinusoidal components to reveal the frequency composition of the signal. It can be expressed as follows:

(1) $$X\left(f\right)=\int_{-\infty }^{\infty }x\left(t\right)e^{-j2\pi ft}dt$$

where $X\left(f\right)$ represents the frequency-domain signal, indicating the amplitude and phase of the signal’s components at various frequencies.

Variational Mode Decomposition (VMD) is a signal decomposition method based on variational inference, designed to decompose complex signals into a set of intrinsic mode functions (IMFs) with different central frequencies. Given an original signal *x(t)*, the number of modes K (i.e., the number of components to be decomposed) is initialized. By selecting an appropriate K, the original signal is decomposed into multiple IMFs. Each IMF has a relatively narrow bandwidth, and its central frequency is adaptively determined by the VMD algorithm. These IMFs represent different frequency components of the signal, effectively avoiding the mode mixing issues found in traditional methods such as Empirical Mode Decomposition (EMD).

In this paper, the original one-dimensional vibration signal is first divided into fixed windows of length 1024. Then, Variational Mode Decomposition (VMD) is employed to decompose each signal segment into four Intrinsic Mode Functions (IMFs) for extracting multi-scale time-domain features. Meanwhile, Fast Fourier Transform (FFT) is applied to the original signal to obtain its frequency-domain amplitude spectrum, which is used to capture the frequency characteristics of the signal. Finally, these four VMD components and the one-dimensional FFT spectrum are fused in the channel dimension to construct a feature tensor with 5 channels and each channel length of 1024. Each sample has a dimension of [5, 1024], where 5 denotes the fused multi-channel features, and 1024 indicates the time steps. Such samples contain the time-frequency information of the vibration signal.

However, when the number of decomposition layers is too large, it may introduce noise. Therefore, we set different decomposition layer numbers and observe the changes in the central frequencies of each mode under different decomposition levels. We select the decomposition layer number when the central frequencies tend to stabilize, ensuring that the chosen modes can adequately represent the frequency characteristics of the signal while avoiding the noise introduced by over-decomposition. By observing and analyzing the central frequency variations across three datasets, we found that when K = 4, the central frequency variations consistently tend to stabilize.

**Figure 2:**
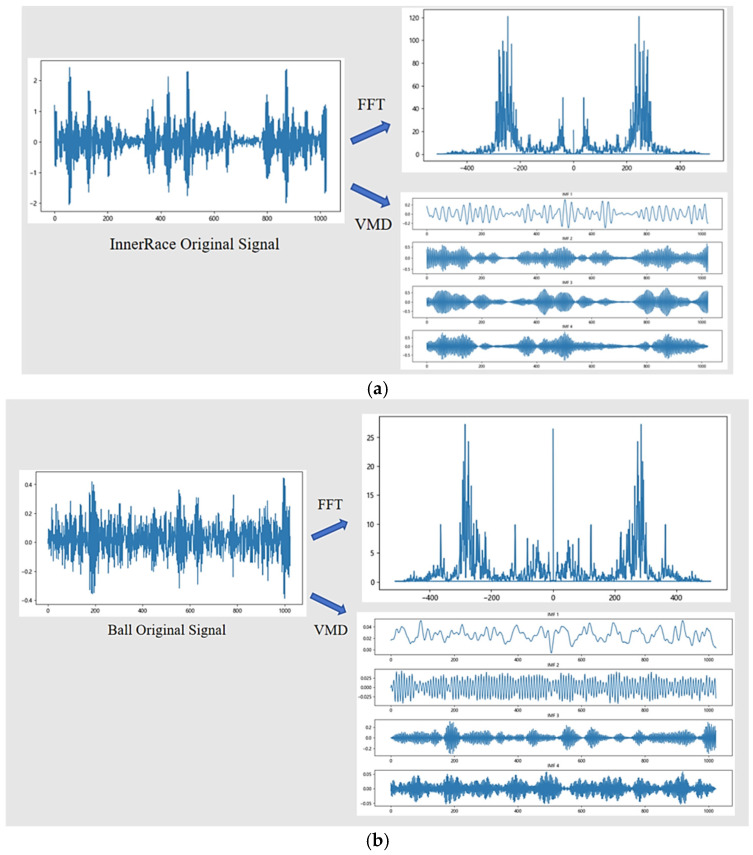
Data Augmentation Process. (**a**) Inner Race (**b**) Rolling Element Faults.

For example, using the Case Western Reserve University (CWRU) bearing dataset, we take the inner race fault and outer race fault signals with a fault size of 0.021 inches as a case study. The sample processing procedure is illustrated in Figure 2.

The original vibration signals from inner race faults and rolling element faults do not exhibit significant differences in the time domain. By applying the FFT transformation, the frequency-domain features of the original signals are obtained. Meanwhile, the VMD transformation extracts deeper-level representations from the time-domain information. The results show that the FFT and VMD representations differ significantly between different fault types. By combining FFT and VMD transformations, this study effectively captures a joint representation of multi-level information from the signals, improving the feature representation and diagnostic accuracy.

### 2.3. Spatiotemporal Feature Extraction

In this study, the preprocessed samples are input into a convolutional network to extract the local spatial features of time series data. The convolutional network is implemented by stacking three identical convolutional blocks, each consisting of a convolutional layer, batch normalization layer, SE layer, max-pooling layer, and dropout layer. The parameter configuration for each layer is shown in Table 1, with the convolutional layers having outchannels of 32, 64, and 128, respectively. In each convolutional block, the input sequence is first processed by a one-dimensional convolution:

(2) $$C_{ij}^{l}=\phi \left(k_{n\times 1}^{j}*x_{i:i+n}^{i}+b_{ij}\right)$$

where, $k_{n\times 1}^{j}$ represents the *j*-th kernel, $x_{i:i+n}^{i}$ is the *i*-th input, $b_{ij}$ is the bias, ϕ is the activation function, and $C_{ij}^{l}$ is the *i*-th feature point of the *j*-th kernel in the *i*-th convolutional layer. Next, the data go through batch normalization and the nonlinear activation function ReLU. Batch normalization is used to stabilize the feature distribution and accelerate convergence, while the nonlinear activation function ReLU is used to enhance the network’s expressive power.

Finally, the channel attention mechanism is used to dynamically adjust the importance of the feature channels after convolution, strengthening key patterns. The pooling layer performs downsampling, further extracting significant features while reducing computational complexity. Dropout is then applied to prevent overfitting and improve the network’s generalization capability.

At the same time, to capture the global temporal features in sequential data, the preprocessed samples are input into a Bidirectional Long Short-Term Memory (BiLSTM) network for feature extraction. BiLSTM is an extension of the traditional Long Short-Term Memory (LSTM) network. Unlike conventional LSTMs, which can only make predictions based on the forward historical information of the input sequence, BiLSTM combines two LSTM models: one processes the input sequence in the forward direction (from past to future), and the other processes it in the reverse direction (from future to past) [27]. This bidirectional structure enables the model to learn information from both past and future contexts, making it particularly effective for tasks where contextual information significantly influences the prediction results.

Once the preprocessed samples are fed into the network, the forward LSTM processes the sequence from left to right, generating a sequence of hidden states, while the backward LSTM processes the sequence from right to left, producing another sequence of hidden states. At each time step, the hidden states from the forward and backward LSTMs are combined to form the final output hidden state. By integrating information from both directions, BiLSTM provides a more comprehensive understanding of the sequential data.

### 2.4. Attention Mechanism

The principle of the attention mechanism is to adaptively assign weights to obtain more discriminative features. The cross-attention mechanism is used to fuse multi-modal spatiotemporal features extracted from parallel networks. The query vector *Q* is obtained through SECNN, and the key vector *K* and value vector V are derived from BiLSTM. First, the similarity between the query *Q* and the key *K* is computed:

(3) $$A=\frac{QK^{T}}{\sqrt{d_{k}}}$$

Here, *Q* comes from the query source, while *K* comes from the value source. The similarity between the query and each key in the value source is computed to obtain the attention matrix *A*. Next, a softmax operation is applied to the attention matrix, yielding the normalized attention weights. Finally, a weighted sum of the values *V* is computed to obtain the final output representation, which is the result of the cross-attention calculation based on the query source and value source:

(4) $$\alpha =Softmax\left(A\right)$$

(5) Z=αV

**Figure 3:**
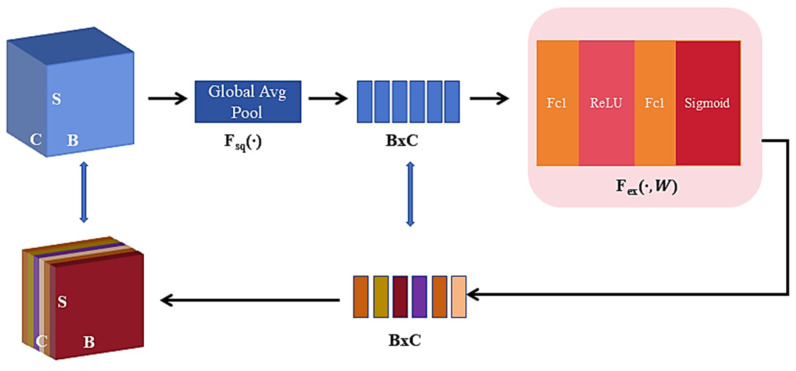
Schematic Diagram of the Working Principle of SE.

The Squeeze-and-Excitation (SE) module, a type of channel attention mechanism, is used to determine the importance of data channels [28], and the SE module in the proposed model is shown in Figure 3. Through global average pooling, the spatial information of each channel is compressed into a scalar along the S (time series length) dimension. Then, a fully connected layer and activation function map the global features of each channel to a lower-dimensional space, compressing the weight of each channel into the range of 0 to 1. Attention weights are then assigned to each channel, enabling the extraction of deep-level features from the time-frequency input. Finally, the features are restored to their original dimensionality.

### 2.5. Objective Function

The objective function of the proposed model consists of two parts: (1) the classification error of labeled data in the source domain, and (2) the domain distribution error obtained using MKMMD [29] after feature extraction for both source and target domain data. To balance the diagnostic accuracy of the source and target domains, the following training strategy is adopted: During the early stage of training, only the source domain data is used for classification, focusing on minimizing classification error. The classifier is optimized using the classification loss function. In the later stage of training, the distribution loss function is introduced to align the features of the source and target domains, thereby reducing domain discrepancy.

In the early stage of training, the classification loss is calculated using the cross-entropy loss function. The formula for cross-entropy loss is as follows:

(6) $$L_{\mathrm{CrossEntropy}}=-\frac{1}{N}\sum_{i=1}^{N}\sum_{c=1}^{C}y_{i,c}log{\hat{p}}_{i,c}$$

where *N* is the number of samples, *C* is the number of classes, $y_{i,c}$ represents the true label of the *i*-th sample, and ${\hat{p}}_{i,c}$ is the predicted probability for the *i*-th sample.

In the later stage of training, MKMMD loss is introduced. By employing multiple Gaussian kernel functions, data points in the original space are mapped to a high-dimensional latent space, enabling linearly inseparable data in the original space to become linearly separable in the high-dimensional space. This nonlinear mapping capability makes it well-suited for complex classification or regression tasks. The formula for each Gaussian kernel and the multi-kernel Gaussian kernel are as follows:

(7) $$k_{i}\left(x,y\right)=exp\left(-\frac{∥x-y{∥}^{2}}{2\times {{(bandwidth}_{i})}^{2}}\right)$$

(8) $$K\left(x,y\right)=\sum_{i=1}^{\mathrm{kernel}.\mathrm{num}}k_{i}\left(x,y\right)$$

where x and y represent different samples, and $∥x-y{∥}^{2}$ is the squared Euclidean distance between the two samples. kernel.num represents the number of Gaussian kernels. The kernel matrix *K* has a shape of *(N + M, N + M)*, where *N* is the number of samples in the source domain and *M* is the number of samples in the target domain. The top-left section of the matrix represents the correlation between source domain samples, while the bottom-right section represents the similarity between target domain samples. The bottom-left and top-right sections represent the similarities between the source and target domain samples, respectively. The bandwidth is calculated using the following formula:

(9) $$bandwidth=\frac{1}{nm}\sum_{i=1}^{n}\sum_{j=1}^{m}∥x_{i}-y_{j}{∥}^{2}$$

(10) $${Bandwidth}_{i}=bandwidth\times {\left(kernelmul\right)}^{i},\;i=0,1,\ldots ,P$$

Among them, n and m represent the number of samples in the source domain and target domain, respectively. $x_{i}$ is the feature vector of the *i*th sample in the source domain, and $y_{j}$ represents the feature vector of the *j*th sample in the target domain. ${∥x}_{i}-y_{j}{∥}^{2}$ is the squared Euclidean distance between the source and target domain samples. kernelmul controls the growth rate of the Gaussian kernel bandwidth, and bandwidth is the initial bandwidth.

The formula for calculating MKMMD and the final objective function is as follows:

(11) $$L\left(\theta_{f},\theta_{c}\right)=L_{\mathrm{CrossEntropy}}\left(\theta_{f},\theta_{c}\right)+\lambda {MKMMD}^{2}\left(\theta_{f}\right)$$

(12) $$\begin{array}{c} {MKMMD}^{2}=\frac{1}{N^{2}}\sum_{i,j\epsilon Source}K(x_{i},x_{j})+\frac{1}{M^{2}}\sum_{i,j\epsilon Target}K(y_{i},y_{j})-\frac{2}{NM}\sum_{i\epsilon Source,j\epsilon Target}K(x_{i},y_{j}) \end{array}$$

Here, N is the number of samples in the source domain, and M is the number of samples in the target domain. $K\left(x_{i},x_{j}\right)$ represents the kernel function between data points in the source domain, $K\left(x_{i},y_{j}\right)$ represents the kernel function between data points in the source and target domains, and $K\left(y_{i},y_{j}\right)$ represents the kernel function between data points in the target domain. $\theta_{f}$ represents the parameter set of the feature extractor, and $\theta_{c}$ represents the parameter set of the classifier. During training, the chain rule is used to compute the gradient of the loss with respect to each parameter layer by layer. The Adam optimizer is then employed to update the parameters of each network layer, aiming to minimize the total loss $L\left(\theta_{f},\theta_{c}\right)$.

## 3. Experimental Study and Analysis

To validate the effectiveness of the STFDAN framework, we conducted comparative experiments on three publicly available datasets against several traditional models. Additionally, to evaluate the impact of each individual component of the model on its diagnostic performance, we performed ablation experiments using the Case Western Reserve University (CWRU) bearing dataset and the Jiangnan University (JNU) bearing dataset.

### 3.1. Data Description and Processing Methods

All experiments in this study were conducted using the PyTorch 1.4 framework. The specific hardware configuration includes an RTX 3080Ti GPU, an Intel i9-10980XE processor, 125.5 GiB of RAM, and a 64-bit Linux operating system.

In the comparative experiments, we primarily tested the proposed model on the following three publicly available bearing datasets. A description of these datasets is provided below:

A. **Case Western Reserve University Bearing Fault Dataset (CWRU)**

**Figure 4:**
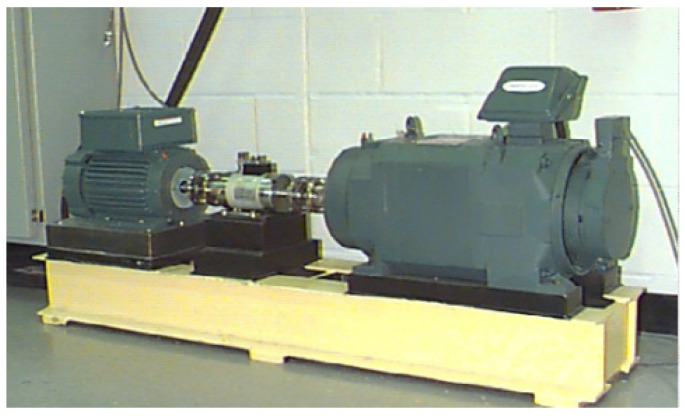
CWRU rolling bearing test rig.

The CWRU dataset is one of the most widely recognized and authoritative publicly available datasets for bearing fault diagnosis [30]. Therefore, this study uses the CWRU dataset as the primary data source for model evaluation. The bearing model used in the dataset is SKF6205, and the fault data collection test rig is shown in Figure 4.

The experimental setup is as follows: the signal acquisition frequency is set to 12 kHz, and the motor load is 0 HP, 1 HP, 2 HP, and 3 HP, corresponding to different rotational speeds. These different speeds represent different operating conditions, or tasks. The corresponding relationships are shown in Table 2. Vibration signals under different migration tasks are obtained by collecting vibration signals from the acceleration sensor on the drive-end bearing. For each migration task, there are 10 categories with corresponding labels from 0 to 9, including one healthy state (NA) and three types of faults. The three types of faults are divided into 9 categories based on fault location and severity. The fault locations include inner race fault (IF), outer race fault (OF), and rolling element fault (BF). Based on the severity of the faults, they are further divided into light fault (0.007 inches), medium fault (0.014 inches), and severe fault (0.021 inches). The corresponding relationships are shown in Table 3. The original vibration signal is sampled with a window length of 1024 without overlap. After applying the data augmentation method introduced in Section 2.2 to the samples, the data is split into training, validation, and test sets in a 3:1:1 ratio. The final distribution of the generated samples is shown in Table 4.

B. **Jiangnan University Bearing Fault Dataset (JNU)**

**Figure 5:**
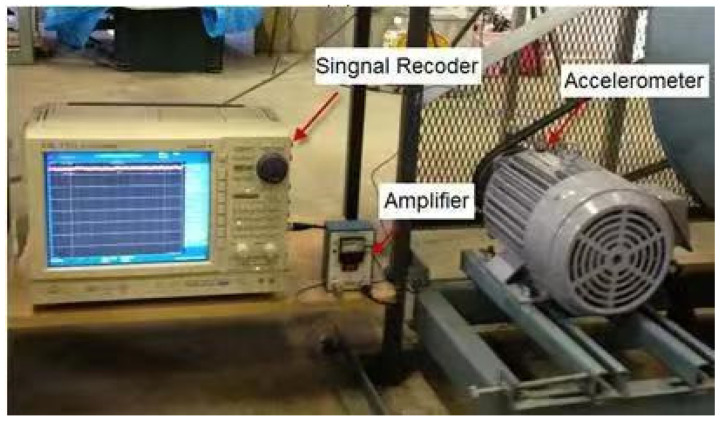
JNU rolling bearing test rig [31].

The JNU dataset is a bearing fault dataset obtained from Jiangnan University [31]. The experimental setup uses a PCB MA352A60 accelerometer to collect vertical vibration signals. The bearing failure test rig at Jiangnan University is shown in Figure 5.

The sampling frequency is 50 kHz, with rotational speeds of 600 r/min, 800 r/min, and 1000 r/min. Each speed corresponds to a different transfer task, as shown in Table 5. In each transfer task, there are four fault types: inner race fault (IF), outer race fault (OF), rolling element fault (BF), and normal state (NA). The corresponding labels and fault mappings are shown in Table 6. The processing method is identical to that used for the CWRU dataset. Finally, the sample set is divided into training, validation, and test sets in a 3:1:1 ratio.

C. **Southeast University Bearing Fault Dataset (SEU)**

The Southeast University bearing dataset is a publicly available dataset provided by Southeast University [32]. This dataset consists of two sub-datasets: one for bearings and one for gears. In this study, only the bearing dataset is used to evaluate the performance of the proposed model. The bearing dataset includes two operating conditions with different rotational speeds and loads: 20 Hz-0 V and 30 Hz-2 V. These two conditions are treated as different transfer tasks, labeled as 0 and 1. In each condition, there is one healthy state (NA) and four fault types. The four fault types are roller fault (BF), inner race fault (IF), outer race fault (OF), and composite fault (CF). The composite fault refers to the simultaneous occurrence of both inner and outer race faults. The corresponding classification categories and label assignments are shown in Table 7. Similar to the data processing methods used for the two datasets mentioned above, vibration data from the eight channels collected is processed. For the subsequent experiments, vibration data in the direction of the planetary gear x (channel 2) is selected.

### 3.2. Impact of Weight λ and Noise on Model Performance

#### 3.2.1. Impact of Weight λ on Model Performance

**Figure 6:**
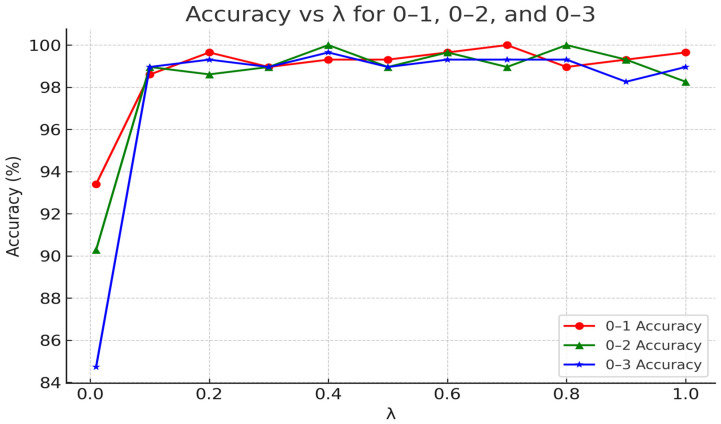
The Impact of Weight on Model Performance.

To illustrate the impact of hyperparameters on the model’s performance, based on the three transfer tasks of 0–1, 0–2, and 0–3 on the CWRU dataset, we discuss the influence of the weight λ of MKMMD on the model’s performance by comparing the model’s test performance under different hyperparameter settings. By setting different weight values and observing the changes in the model’s test performance, we set the range of λ as 0.01, 0.1, and up to 1 with an interval of 0.1 to study the influence of the weight parameter on the model’s test performance. The impact of the weight parameter on model test performance is illustrated in Figure 6.

We found that when the MKMMD loss weight was set to 0.01, the weight value was too small to effectively align the distribution differences between the source and target domains. As a result, the diagnostic accuracy in the target domain remained below 94%. When the weight was set between 0.1 and 0.4, the model’s test accuracy remained above 98%, meeting practical application requirements, indicating that the introduction of the MKMMD loss had a positive effect. However, when the weight was set between 0.8 and 1, we observed a slight overall decline in accuracy. Therefore, the model achieves better and more stable test performance when the MKMMD loss weight is set between 0.4 and 0.8.

#### 3.2.2. Impact of Noise on Model Performance

Another important factor that affects the model’s ability to accurately diagnose the bearing condition under complex operating conditions is the interference effect of noise, especially in the case of low amplitude fault signals and strong noise, the noise mixing makes it difficult for the bearing data to clearly reflect the bearing condition information. An important index used to measure the strength of noise in the signal is the signal-to-noise ratio, which is calculated by the following formula:

(13) $${SNR}_{dB}=10\log_{10}\left(\frac{P_{signal}}{P_{noise}}\right)$$

where $P_{signal}$ is the signal power and $P_{noise}$ is the noise power. The size of the signal-to-noise ratio is inversely proportional to the intensity of the noise, and when SNR=0 dB, the energy intensity of the signal and the noise are equal.

**Figure 7:**
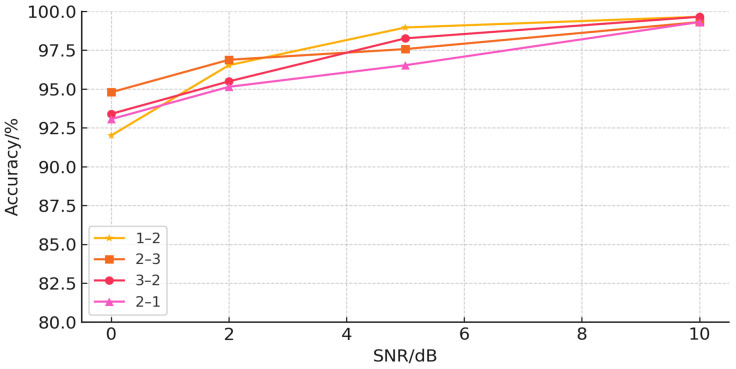
The Impact of noise on Model Performance.

We tested the sensitivity of the model to different intensities of noise by adding different intensities of Gaussian noise to the target domain data in the 1–2, 2–3, 3–2, and 2–1 migration tasks of the CWRU dataset, with signal-to-noise ratios taking the values of 0 dB, 2 dB, 5 dB, and 10 dB. The results are shown in Table 8 below, and their corresponding line plots are shown in Figure 7.

As can be seen from Figure 7, although the diagnostic accuracy gradually decreases with the enhancement of the noise intensity, the diagnostic accuracies of the four migration tasks remain above 92% when the energy intensity of the signal and the noise are equal, which meets the needs of practical engineering applications and indicates that the model has good noise immunity. However, improving the noise resistance of the model is still a continuing research direction in future work.

### 3.3. Comparative Experiments

This section presents comparative experiments based on the three publicly available datasets mentioned in Section 3.1. Six widely used transfer learning methods are selected for comparison with the proposed model. For the fairness of comparison, we conduct comparative experiments on the same experimental computing platform, and the hyperparameter settings are kept consistent. Among them, the learning rate is set to 0.0003 and the batch size is set to 32. These methods include the machine learning-based Support Vector Machine (SVM) [33], Domain Adaptation with Deep Convolutional Networks (DDC) [34], Deep Domain-Convolutional Neural Networks (DFCNN) [35], Domain-Adversarial Neural Network (DANN) [36], Correlation Alignment (CORAL) [37], and Multi-Kernel Maximum Mean Discrepancy (MKMMD).

**Figure 8:**
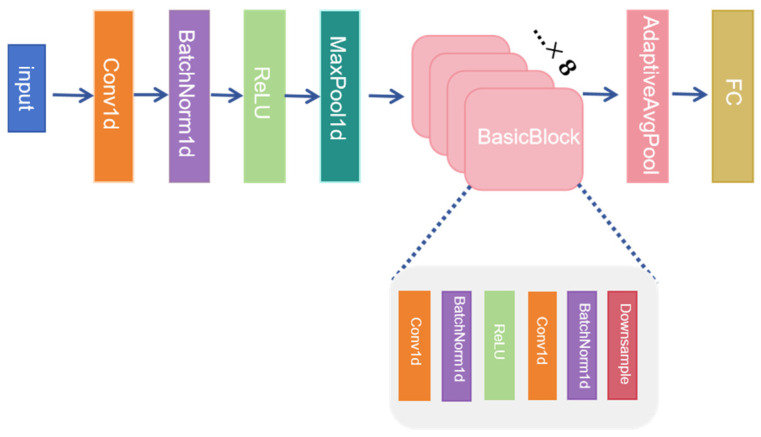
Network Architecture of ResNet-18.

The SVM uses an RBF kernel function with parameters C = 100 and γ = 0.01. The DDC introduces an adaptive layer and an additional domain confusion loss to learn domain invariant representations. DFCNN converts 784 samples to grayscale images for training to extract deeper fault features. To ensure that the comparative algorithms are representative, the feature extractors used in the DANN and CORAL methods are based on ResNet-18 [38], and the MKMMD methods are categorized into ResNet-18 based feature extractor methods (RMKMMD) and Transformer [39] based feature extractor methods (TMKMMD). ResNet-18 is particularly effective for processing time series data. The network structure is shown in Figure 8.

A. **Comparative Experiments on the Case Western Reserve University (CWRU) Dataset**

In the bearing dataset experiments from Case Western Reserve University, 12 variable operating condition fault diagnosis tasks are involved. To conveniently represent the transfer tasks, “a–b” is used to denote the transfer task from source domain “a” to target domain “b”. To verify the superiority of the proposed STFDAN model, comparisons are made with the seven different fault diagnosis methods mentioned earlier. In addition, we also evaluated whether the computational cost of the proposed STFDAN model is reasonable by comparing the average training time (Time) across the 12 transfer tasks. The unit of training time is consistently measured in seconds (s) in the subsequent experiments. The diagnostic results of the different methods for the 12 cross-domain tasks are shown in Table 9.

As can be seen from Table 9, STFDAN outperforms the other seven compared methods in most of the transfer tasks. The STFDAN model extracts features using parallel SECNN and BiLSTM architectures and fuses the spatiotemporal features through a cross-attention mechanism to obtain the final feature representation. Compared to the RMKMMD approach, which uses ResNet-18 as a feature extractor, STFDAN shows significant advantages in 9 out of 12 transfer tasks. Notably, STFDAN outperforms RMKMMD by 6.25% in the 3–0 transfer task. Compared with the TMKMMD method, which uses Transformer as the feature extractor, STFDAN demonstrates a more obvious superiority, not only in terms of higher accuracy but also in terms of less training time. Overall, this indicates that STFDAN is more advantageous in feature extraction when dealing with complex transfer conditions.

In addition, STFDAN achieved higher accuracies in eight transfer tasks compared to DANN and CORAL. In the 3–0 transfer task, the accuracy of STFDAN is 7.03% to 7.43% higher than that of DANN and CORAL. This indicates that STFDAN does not fully utilize its advantages in transfer tasks with small feature differences (e.g., low-to-high-speed faulty data transfers), but in complex transfer tasks, especially in the 3–0 task, STFDAN shows significant advantages. Overall, STFDAN maintains an accuracy of over 98% in all transfer tasks, which demonstrates its stability. It performs particularly well in the 3–0 transfer task, which indicates its suitability for complex transfer learning scenarios.

By comparing the three models—RMKMMD, DANN, and CORAL—which all use ResNet-18 as the feature extractor, it is evident that DANN exhibits very stable performance across most tasks, especially in tasks 0–1, 2–3, and 3–2, where its accuracy is close to or reaches 100%. This indicates that DANN has strong adaptability, even in transfer tasks where there are significant differences between the source and target domains. CORAL performs exceptionally well in tasks where the difference between the source and target domains is smaller or where feature alignment is more evident, particularly in tasks 0–1, 2–3, and 1–2. It effectively reduces domain discrepancies by aligning the covariance matrices of the source and target domains. RMKMMD performs well in tasks 3–1, 3–0, and 3–2, achieving higher accuracy than the other two models. This suggests that RMKMMD is more effective in low-speed to high-speed transfer tasks, where the distribution differences between the source and target domains are more pronounced.

From the perspective of computational cost, the comparative analysis of the methods in Table 9 shows that our proposed STFDAN model requires less training time with higher average accuracy compared to DANN, CORAL, RMKMMD and TMKMMD. Specifically, compared to the RMKMMD method that uses ResNet-18 as a feature extractor, our proposed STFDAN model takes only 121 s, which is 25 s less than RMKMMD. Compared with the TMKMMD method that uses Transformer as a feature extractor, the TMKMMD method requires about three times the training time of our proposed STFDAN model, with a larger time cost. This indicates that the parallel network architecture proposed by STFDAN has lower computational overhead than ResNet-18 and is more suitable for industrial applications. DFCNN does not involve the alignment process of source and target domains, but only trains the source domain data and applies the model directly to the target domain for testing, resulting in shorter training time. Meanwhile, DDC is based on the AlexNet architecture, which is relatively simple and thus has a shorter training time than STFDAN. However, the average accuracy of STFDAN is 15.26% higher than that of DDC. Considering all the factors, STFDAN has better overall performance.

Furthermore, by analyzing the experimental results, we observed a common pattern across all transfer learning methods: when the source domain consists of low-speed datasets and the target domain consists of high-speed datasets, the performance of transfer learning is consistently lower than that of other transfer tasks. This phenomenon occurs because high-speed features are more complex, containing more high-frequency components and intricate vibration patterns.

When transferring from low-speed feature recognition to high-speed feature recognition, certain challenges arise, leading to a slight decrease in accuracy in transfer tasks 2–0 and 3–0. Additionally, the feature distribution gap between the source and target domains in these tasks is relatively large, especially in the 3–0 task. Conducting feature alignment between the source and target domains using an unsupervised approach becomes more challenging, introducing difficulties in feature alignment.

**Figure 9:**
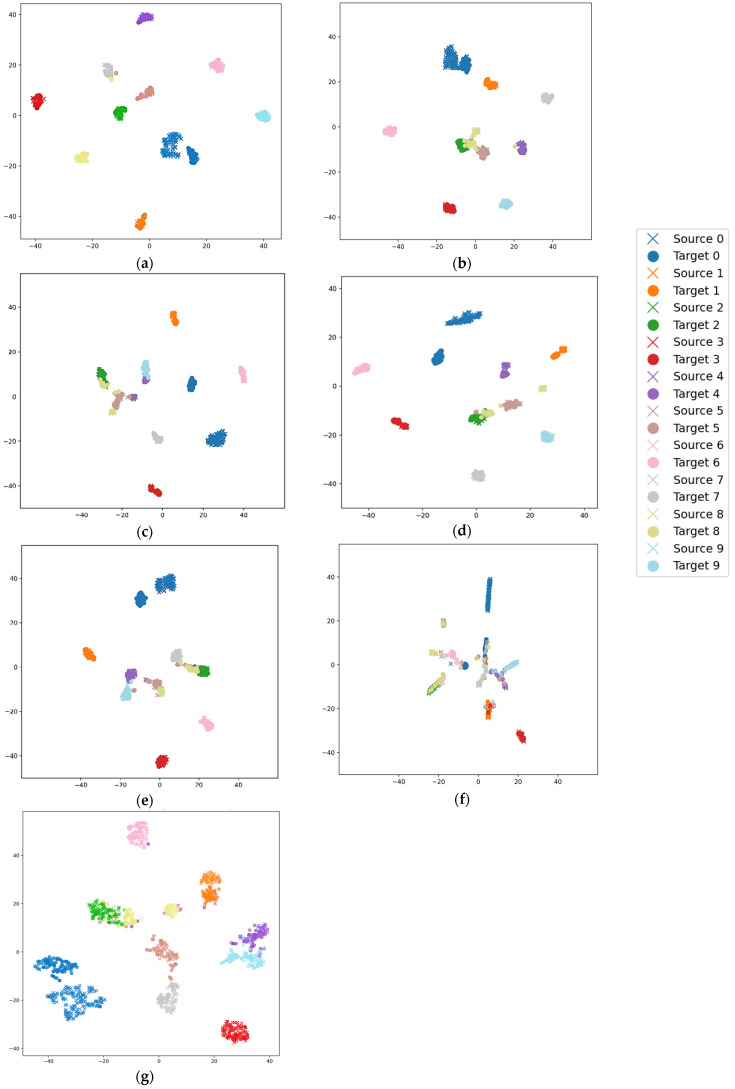
t-SNE Feature Visualization for Migration Task 3–0. (**a**) STFDAN; (**b**) RMKMMD; (**c**) DANN; (**d**) CORAL; (**e**) DFCNN; (**f**) DDC; (**g**) TMKMMD.

In the 3–0 migration task, it is challenging to effectively align the two domains because of the large difference in rotation speed between the source domain (low speed) and the target domain (high speed). In order to visualize the feature extraction capability of the proposed model, we take the 3–0 migration task as an example and apply the t-distributed stochastic neighborhood embedding (t-SNE) algorithm to visualize the features extracted by the DDC, DFCNN, DANN, CORAL, RMKMMD, TMKMMD and STFDAN methods. The t-SNE feature visualization for each method is shown in Figure 9.

From the feature map in Figure 9a, it can be observed that the inter-domain distance between source domain samples and target domain samples is relatively small, while within each domain, the distances between different categories are relatively large. The experimental results indicate that the proposed model can effectively align the source and target domains and correctly classify faults.

**Figure 10:**
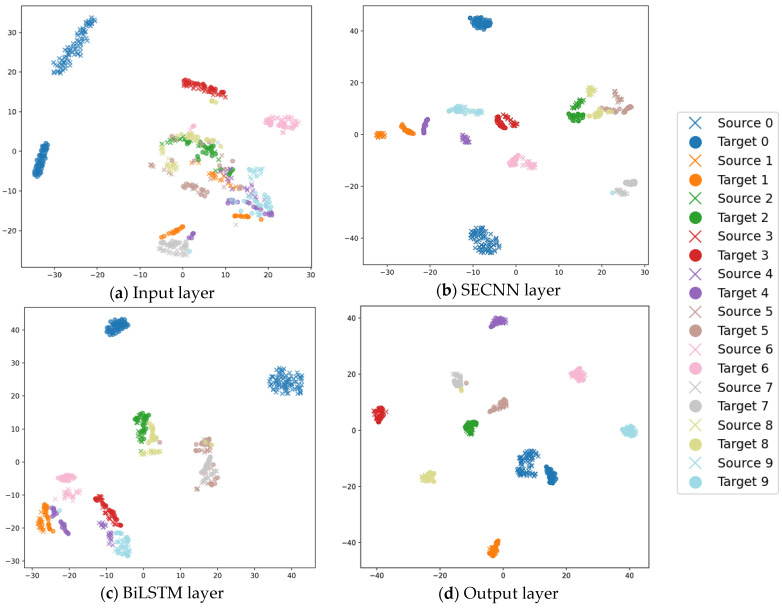
Feature Extraction Visualization Based on the STFDAN Model Structure.

To visualize the feature extraction process in the STFDAN model, we use t-SNE plots to analyze the extracted features for the 3–0 transfer task from a network architecture perspective. The extracted features from the SECNN layer, BiLSTM layer, and the final fully connected layer are visualized, as shown in Figure 10. Figure 10a illustrates the feature distribution of the source and target domain test samples before being input into the model. Figure 10b shows the output features from the SECNN layer. Figure 10c presents the output features from the BiLSTM layer. Figure 10d depicts the output features from the final fully connected layer after training.

From these visualizations, we observe that both SECNN and BiLSTM have certain feature extraction capabilities. As the network deepens, the overlap between different fault categories in the source and target domains significantly decreases, and the feature distribution of the same category becomes more concentrated.

**Figure 11:**
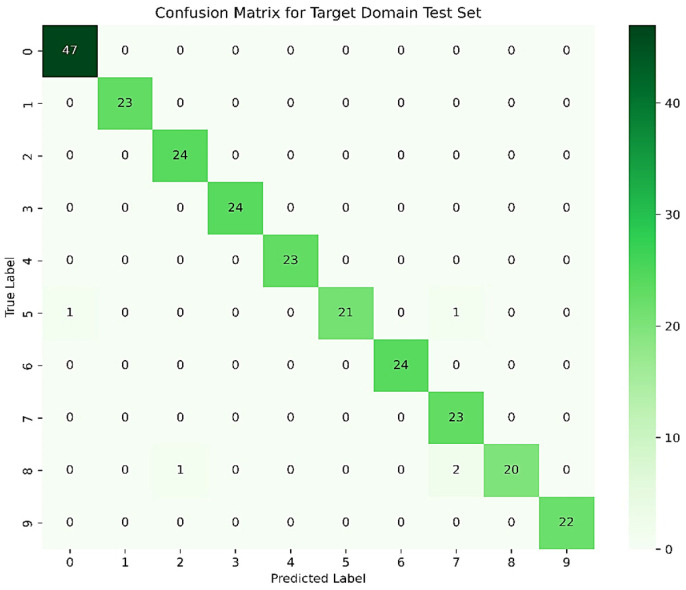
Confusion Matrix for Migration Task 3–0.

The confusion matrix for the 3–0 transfer task is shown in Figure 11. By observing the confusion matrix, it can be seen that almost all samples in the target domain test set are correctly classified, with only 5 samples being misclassified. This further validates that the proposed model exhibits superior performance compared to other methods.

B. **Comparative Experiments on the Jiangnan University (JNU) Dataset**

In the experiments with the Jiangnan University dataset, there are three operating conditions, resulting in a total of six transfer tasks. Similar to the comparison experiments with the Case Western Reserve University dataset, we compare the accuracy and average training time (Time) of the proposed STFDAN model with the mentioned different fault diagnosis methods.

**Figure 12:**
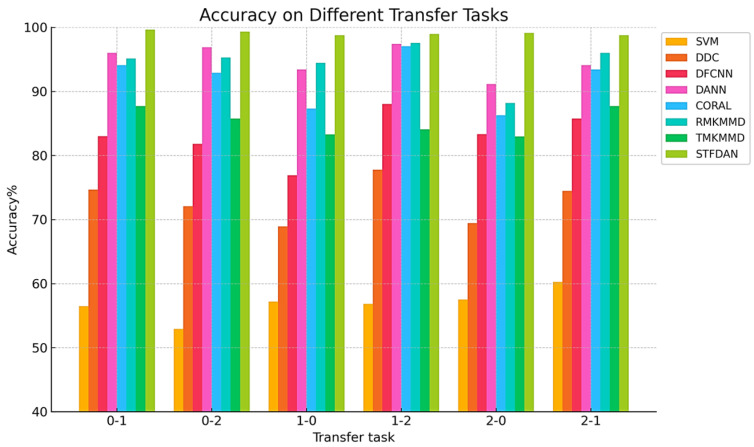
Experimental Results on the JNU Dataset.

The basic parameters are kept consistent, and the experiments are conducted on the same computational platform. The diagnostic accuracies for the six transfer tasks across different methods are shown in Table 10, and the corresponding histograms are presented in Figure 12.

The experimental data show that the proposed model still achieves high accuracy on the JNU dataset. Compared with the CWRU dataset, the advantage of our proposed model on the JNU dataset is more obvious. The average accuracy is 4.28% higher than the highest accuracy among the other six methods. The diagnostic accuracies for each transfer task ranged from 98.78% to 99.65%, indicating that STFDAN has higher adaptability and stronger generalization ability compared to other methods. Comparison in terms of time cost reveals that although DDC and DFCNN require less time, their average fault diagnosis accuracies are only between 72.89% and 83.14%, with a relatively high risk of missed and incorrect tests in practical applications. Meanwhile, compared with our proposed STFDAN model, DANN, CORAL, RMKMMD and TMKMMD require more training time but have lower accuracy rates. These results further demonstrate the significant value of the proposed STFDAN model in practical engineering applications.

**Figure 13:**
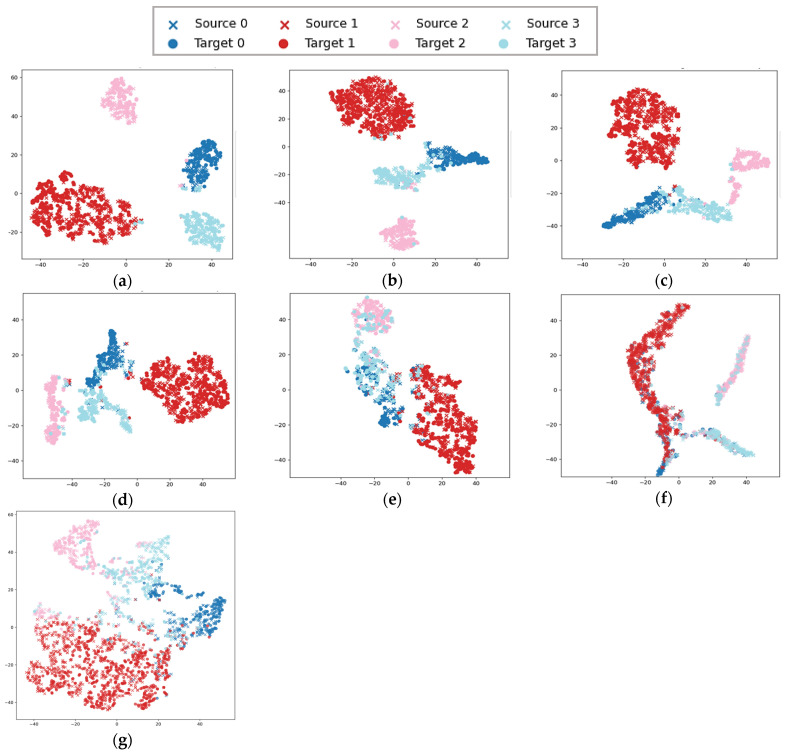
t-SNE Feature Visualization for Migration Task 0–2. (**a**) STFDAN; (**b**) RMKMMD; (**c**) DANN; (**d**) CORAL; (**e**) DFCNN; (**f**) DDC; (**g**) TMKMMD.

To visually demonstrate the contribution of the STFDAN model to migration fault diagnosis, we also perform t-SNE feature visualization for the comparative experiments on the JNU dataset. The results are shown in Figure 13. To make the migration effect more intuitive, we label the source and target domain samples with different symbols, using the same color for the same class in both domains. By observing Figure 13, it is evident that the STFDAN model outperforms other methods. This is primarily reflected in the fact that not only are the source and target domain data completely aligned in feature space, but the distance between different classes is also large, with samples of the same class clustered together. This indicates that the migration effect is excellent, and most samples are correctly classified.

**Figure 14:**
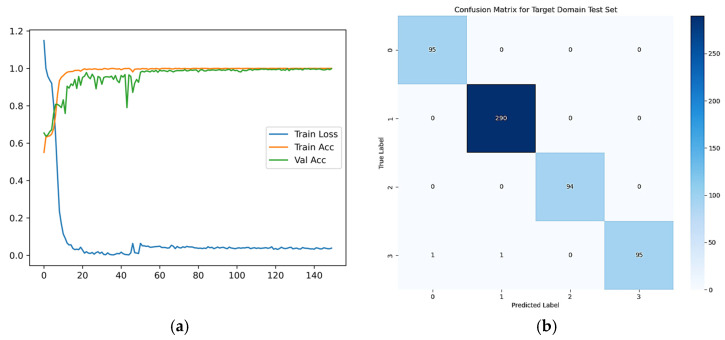
Migration Task 0–1 on the JNU Dataset. (**a**) Accuracy and Loss Curves; (**b**) Confusion Matrix.

Additionally, to further assess the convergence speed and learning ability of the STFDAN model, we plot the accuracy and loss curves, as well as the confusion matrix for Task 0–1, to better observe the model’s status during the training process, as shown in Figure 14. The curves include the accuracy curve for the source domain training set, the accuracy curve for the target domain validation set, and the loss curve for the entire training phase. The loss curve for the first 50 epochs records the classification loss for the source domain training set, while the subsequent 100 epochs record both classification loss and domain adaptation loss. As shown in Figure 14a, the accuracy and loss values stabilize after the 10th epoch, indicating that the model exhibits strong stability. After the 50th epoch, with the addition of domain alignment loss, the accuracy curve for the target domain validation set gradually stabilizes and approaches 100%. This suggests that the STFDAN model effectively captures the features of the data and learns meaningful features. In the confusion matrix of Figure 14b, the predicted labels are almost identical to the true labels, with the majority of samples correctly diagnosed, as shown along the diagonal.

C. **Comparative Experiments on the Southeast University (SEU) Dataset**

There are two migration tasks in the comparison experiment on the Southeast University dataset. We again used the seven methods mentioned earlier for comparison, and the results are shown in Table 11. On the Southeast University dataset, the STFDAN model shows excellent performance. The highest average accuracy of the other seven methods is only 76.00%, while the STFDAN model achieves an accuracy of 99.22%, which is 23.22% higher than 76.00%. In addition, the STFDAN model significantly outperformed the other methods in both migration tasks.

The SEU dataset has more complex fault modes, which not only include single faults such as inner race, outer race, and rolling element faults, but also composite fault modes involving both inner and outer races. Additionally, the SEU dataset consists of data collected at different frequency levels (20 Hz-0 V and 30 Hz-2 V), so the data distribution differences in the SEU dataset are larger compared to the other two datasets, which were collected at the same frequency. When using other models, the feature extraction capability is limited, making it challenging to perform feature alignment with unsupervised transfer learning methods. On the other hand, the STFDAN model processes the input data from different perspectives and, through its parallel network structure, separately extracts temporal and spatial features before fusing them. This approach enables the extraction of more comprehensive feature representations from input data with large distribution differences, which is more beneficial for subsequent feature alignment. This further demonstrates that the proposed STFDAN model is more capable of handling complex transfer tasks.

In the comparison of computational overhead, we find that although DDC and DFCNN take less time than STFDAN, the average accuracy of STFDAN is 25.94% higher than DFCNN and 58.75% higher than DDC. The DANN, CORAL and RMKMMD using ResNet-18 as a feature extractor and the TMKMMD method using Transformer as a feature extractor all take more time than our proposed STFDAN model, resulting in higher computational overhead with lower accuracy than STFDAN. In conclusion, the STFDAN model achieves higher fault diagnosis accuracy while maintaining relatively low computational overhead, making it valuable in practical applications.

**Figure 15:**
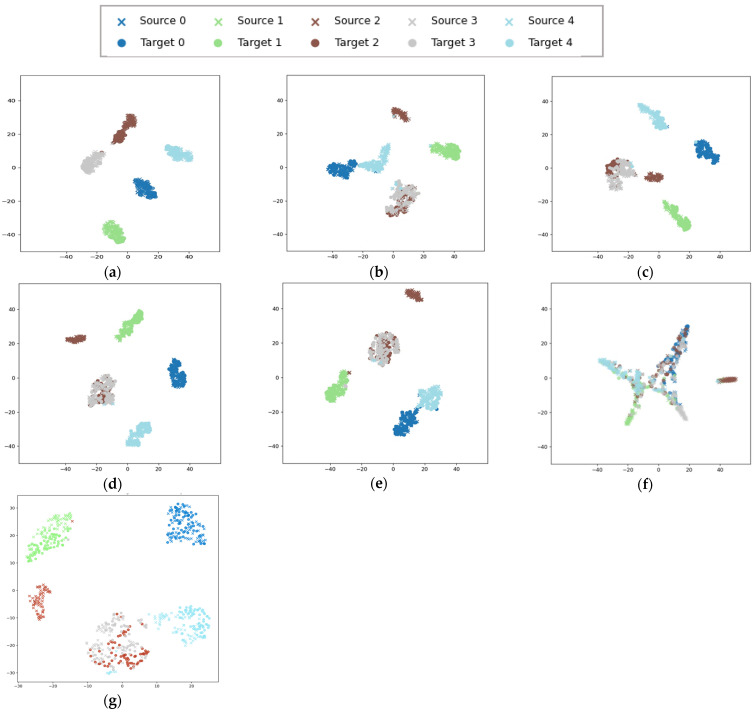
t-SNE Feature Visualization for Migration Task 0–1. (**a**) STFDAN; (**b**) RMKMMD; (**c**) DANN; (**d**) CORAL; (**e**) DFCNN; (**f**) DDC; (**g**) TMKMMD.

To present the experimental results more intuitively, we performed feature visualization on the source and target domain test set data for migration task 0–1, as shown in Figure 15. In Figure 15a, both the classification and migration effects are clearly good. In the other five methods, the target domain data for label 2 are significantly different in distribution from the source domain data, and the target domain data are incorrectly classified as label 3. However, in the feature map of our model, almost all samples for label 2 are correctly classified. This further validates the generalization ability and practical applicability of the proposed model.

**Figure 16:**
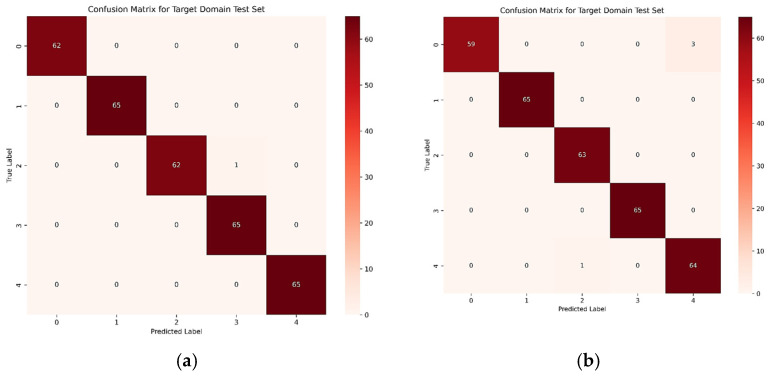
Confusion Matrices for SEU Dataset Migration Tasks. (**a**) 0–1; (**b**) 1–0.

Additionally, we plotted the confusion matrices for the different migration tasks on the SEU dataset to further demonstrate the significant value of the proposed STFDAN model for engineering applications. The confusion matrices for the two migration tasks are shown in Figure 16.

### 3.4. Ablation Experiment

To assess the impact of each module in the STFDAN model on the overall performance, we conducted a series of experiments on the CWRU dataset and the JNU dataset. Based on the STFDAN model, we introduced the following experiments:

(a) Input with only VMD, without FFT.
(b) Removal of the SECNN component, leaving only the BiLSTM.
(c) Removal of the cross-attention mechanism, directly concatenating spatiotemporal features.
(d) Removal of the BiLSTM component, leaving only the SECNN module.
(e) Removal of the SE module.
(f) Input with only FFT, without VMD.
(g) The proposed STFDAN model.

The results of the corresponding ablation experiments are shown in Table 12 and Table 13. By analyzing the above results, the final average classification accuracy of the STFDAN model is improved compared to the other experiments. This indicates that each part of the model contributes to the overall performance to different degrees. In Table 12 and Table 13, although the performance of using only the VMD as input is relatively poor, combining the VMD with the FFT significantly improves the final diagnostic accuracy, which indicates that the FFT contributes significantly to the model.

However, both datasets demonstrate lower overall model performance than the STFDAN model when only the FFT is input, suggesting that the VMD and FFT complement each other and together provide more comprehensive information about the sample. After removing the SE module and the cross-attention mechanism module, we observe a decrease in diagnostic accuracy, suggesting that the SE module plays a role in adjusting the weights of the input information channels, while the cross-attention mechanism is crucial for the fusion of spatiotemporal features. In summary, each module contributes to the final performance, and one is indispensable.

## 4. Conclusions

In this paper, the STFDAN model is proposed to solve the bearing fault diagnosis task under different operating conditions. The network effectively solves the core problems of distribution drift and feature mismatch under changing conditions through parallel spatiotemporal feature extraction, attention-guided feature fusion, and high-dimensional distribution alignment strategies. We conducted comparative experiments on three different publicly available bearing failure datasets to compare the STFDAN model with six other transfer learning methods. Compared to the other methods, the STFDAN model has improved fault classification accuracy under different operating conditions. To validate the usefulness of each module in the model, we conducted ablation experiments on the CWRU and JNU datasets. Through comparison and ablation experiments, we demonstrate that the proposed STFDAN model has good generalization ability and robustness, providing valuable insights for future research.

## Figures and Tables

**Table 1:** Topology of the Convolutional Block.

Layers	Parameters	Activation Function	Output Size
Input	/	/	5 × 1024
Conv1	Kernel: 3 × 5 × out_channels Bias: 32 × 1	/	1024 × 32
BatchNorm1	32 Channels	ReLU	1024 × 32
SEBlock1	32 Channels	Sigmoid	1024 × 32
MaxPool	Kernel: 2, Stride: 2	/	512 × 32
Dropout	Rate: 0.3	/	512 × 32

**Table 2:** CWRU Transfer Tasks.

Load (HP)	0	1	2	3
Speed (rpm)	1797	1772	1750	1730
Task	0	1	2	3

**Table 3:** CWRU Classification Description.

Class Label	0	1	2	3	4	5	6	7	8	9
Location	NA	IF	BF	OF	IF	BF	OF	IF	BF	OF
Size (inches)	0	7	7	7	14	14	14	21	21	21

**Table 4:** CWRU Sample Distribution.

Healthy State	Fault Size	0 HP	1 HP	2 HP	3 HP	All
NA	0	238	472	473	474	1657
IF	0.007 inches	118	119	119	120	476
	0.014 inches	118	118	118	118	472
	0.021 inches	119	118	118	119	474
BF	0.007 inches	119	118	118	118	473
	0.014 inches	118	119	119	119	475
	0.021 inches	119	118	119	119	475
OF	0.007 inches	119	119	118	119	475
	0.014 inches	118	119	118	119	474
	0.021 inches	119	119	119	119	476
All	/	1305	1539	1539	1544	5927

**Table 5:** JNU Transfer Tasks.

Speed (rpm)	600	800	1000
Task	0	1	2

**Table 6:** JNU Classification Description.

Class Label	0	1	2	3
Location	IF	NA	OF	BF

**Table 7:** SEU Classification Description.

Class Label	Location	Description
0	NA	Health
1	BF	Crack in the ball
2	OF	Crack in the outer ring
3	IF	Crack in the inner ring
4	CF	Crack in the outer and inner ring

**Table 8:** Antinoise Experiments Results on the CWRU Dataset.

	1–2	2–3	3–2	2–1
0 dB	92.01%	94.79%	93.40%	93.06%
2 dB	96.53%	96.88%	95.49%	95.14%
5 dB	98.96%	97.57%	98.26%	96.53%
10 dB	99.65%	99.31%	99.65%	99.31%

**Table 9:** Experimental Results on the CWRU Dataset.

Task	SVM	DDC	DFCNN	DANN	CORAL	RMKMMD	TMKMMD	STFDAN
0–1	55.84%	90.08%	95.83%	99.31%	98.26%	97.92%	91.25%	99.65%
0–2	50%	90.08%	99.31%	99.31%	97.22%	99.31%	93.06%	99.31%
0–3	47.25%	81.94%	87.50%	100%	91.32%	99.31%	92.96%	99.31%
1–0	50.19%	82.42%	92.58%	98.05%	98.05%	97.66%	91.84%	99.22%
1–2	49.35%	87.85%	98.26%	99.65%	99.31%	99.65%	88.19%	100%
1–3	54.05%	84.03%	89.24%	99.65%	99.31%	98.96%	91.59%	99.65%
2–0	46.74%	75.34%	91.02%	98.05%	97.66%	97.66%	87.76%	98.26%
2–1	59.09%	85.76%	94.44%	99.65%	98.26%	98.96%	93.68%	99.31%
2–3	55.66%	89.58%	95.49%	99.65%	99.65%	99.65%	86.55%	100%
3–0	41.38%	68.36%	86.72%	91.02%	90.62%	91.80%	83.61%	98.05%
3–1	53.57%	85.42%	87.85%	92.36%	92.36%	99.31%	90.83%	99.65%
3–2	52.27%	88.54%	97.57%	99.31%	98.26%	100%	91.32%	100%
Ave	51.28%	84.11%	92.97%	98.00%	96.69%	98.37%	90.22%	99.37%
Time	-	99 s	85 s	157 s	144 s	146 s	452 s	121 s

**Table 10:** Experimental Results on the JNU Dataset.

Task	SVM	DDC	DFCNN	DANN	CORAL	RMKMMD	TMKMMD	STFDAN
0–1	56.48%	74.65%	82.99%	96.01%	94.1%	95.14%	87.72%	99.65%
0–2	52.90%	72.05%	81.80%	96.88%	92.88%	95.31%	85.76%	99.31%
1–0	57.17%	68.92%	76.91%	93.40%	87.33%	94.44%	83.29%	98.78%
1–2	56.83%	77.78%	88.02%	97.40%	97.05%	97.57%	84.06%	98.96%
2–0	57.51%	69.44%	83.33%	91.15%	86.28%	88.19%	82.95%	99.13%
2–1	60.24%	74.48%	85.76%	94.10%	93.40%	96.01%	87.72%	98.78%
Ave	56.86%	72.89%	83.14%	94.82%	91.84%	94.44%	85.25%	99.10%
Time	-	220 s	207 s	347 s	313 s	322 s	752 s	264 s

**Table 11:** Experimental Results on the SEU Dataset.

Task	SVM	DDC	DFCNN	DANN	CORAL	RMKMMD	TMKMMD	STFDAN
0–1	21.7%	38.44%	77.5%	77.5%	79.37%	76.25%	78.01%	99.69%
1–0	23.75%	42.5%	69.06%	69.06%	70%	69.09%	73.98%	98.75%
Ave	22.73%	40.47%	73.28%	74.06%	74.69%	72.67%	76.00%	99.22%
Time	-	128 s	120 s	198 s	186 s	187 s	573 s	151 s

**Table 12:** Ablation Experiment Results on the CWRU Dataset.

Task	a	b	c	d	e	f	g
0–1	83%	96.53%	96.53%	96.18%	99.31%	99.31%	99.65%
0–2	83.33%	98.61%	96.5%	97.92%	97.57%	99.65%	99.31%
0–3	68.06%	92.36%	94.44%	95.14%	93.75%	97.57%	99.31%
1–0	80.86%	93.75%	90.62%	96.48%	98.83%	99.61%	99.22%
1–2	84.03%	91.67%	96.88%	98.26%	98.96%	100%	100%
1–3	64.24%	87.85%	91.32%	97.92%	99.65%	98.96%	99.65%
2–0	69%	82.42%	96.53%	89.45%	90.23%	90.62%	98.26%
2–1	70.49%	98.26%	89.58%	96.88%	99.31%	99.65%	99.31%
2–3	77.08%	94.79%	97.92%	97.57%	100%	99.65%	100%
3–0	62.50%	85.16%	86.72%	91.02%	94.92%	96.09%	98.05%
3–1	68.06%	79.51%	91.67%	97.57%	99.65%	98.96%	99.65%
3–2	78.82%	96.88%	98.61%	98.26%	99.65%	98.61%	100%
Ave	74.12%	91.48%	93.94%	96.05%	97.65%	98.22%	99.37%

**Table 13:** Ablation Experiment Results on the JNU Dataset.

Task	a	b	c	d	e	f	g
0–1	82.64%	88.19%	76.56%	90.80%	98.44%	98.96%	99.65%
0–2	82.52%	81.08%	67.01%	91.32%	97.74%	95.66%	99.31%
1–0	83.14%	78.99%	80.03%	82.64%	98.09%	96.70%	98.78%
1–2	76.91%	91.32%	86.63%	94.97%	99.13%	98.44%	98.96%
2–0	78.82%	83.33%	82.81%	89.24%	91.15%	91.84%	99.13%
2–1	84.38%	92.01%	86.63%	94.44%	98.26%	98.96%	98.78%
Ave	81.40%	85.82%	79.95%	90.57%	97.14%	96.76%	99.10%

## Data Availability

The data are publicly available.
